# Metabolic Signatures of Air Pollution and Major Adverse Cardiovascular Events in Patients Undergoing Cardiac Catheterization

**DOI:** 10.1016/j.jacadv.2025.102481

**Published:** 2026-01-28

**Authors:** Chang Liu, Duan Wang, Zhihao Jin, Yi-An Ko, Howard H. Chang, Ayman A. Alkhoder, Nisreen Haroun, Yan V. Sun, Vilinh Tran, Dean P. Jones, Arshed A. Quyyumi, Donghai Liang

**Affiliations:** a Department of Epidemiology, Emory University Rollins School of Public Health, Atlanta, Georgia, USA; b Gangarosa Department of Environmental Health, Emory University Rollins School of Public Health, Atlanta, Georgia, USA; c Department of Biostatistics, Emory University Rollins School of Public Health, Atlanta, Georgia, USA; d Division of Cardiology, Department of Medicine, Emory University School of Medicine, Atlanta, Georgia, USA; e Department of Pulmonary, Allergy, Critical Care and Sleep Medicine, Emory University School of Medicine, Atlanta, Georgia, USA

**Keywords:** air pollution, CO, MACE, MWAS, NOx, PM_2.5_

## Abstract

**Background:**

Air pollution is an environmental risk factor for coronary artery disease. The molecular mechanisms linking fine particulate matter (PM_2.5_), nitrogen oxide (NOx), and carbon monoxide (CO) to coronary artery disease prognosis remains unclear.

**Objectives:**

The objective of the study was to investigate the molecular pathways linking air pollution to cardiovascular risk by analyzing the metabolome, focusing on the mediating role of metabolites.

**Methods:**

We analyzed data from the Emory Cardiovascular Biobank, including 244 participants with metabolomic profiling, air pollution data, and a median follow-up of 9.8 (IQR: 4.6-12.2) years. Metabolomic profiling was performed via liquid chromatography-high- resolution mass spectrometry, and pollutants (PM_2.5_, NOx, CO) were estimated based on residence. Linear regression assessed pollutant-metabolite associations, adjusting for demographics and clinical factors. Competing risk models examined major adverse cardiovascular events (MACE), and Cox models evaluated all-cause mortality. Pathway and mediation analyses explored pollution-related metabolites.

**Results:**

Per IQR higher in CO was associated with a 24% higher risk in MACE (subdistribution HR [sHR]: 1.24; 95% CI: 1.03-1.49; *P* = 0.02), whereas PM_2.5_ (sHR: 1.05; 95% CI: 0.86-1.30; *P* = 0.62) and NOx (sHR: 1.19; 95% CI: 0.99-1.44; *P* = 0.068) suggested a modest but not significant elevated risk. Both NOx and CO demonstrated associations with cardiovascular mortality, whereas NOx was associated with congestive heart failure. 1-oleoyl-rac-glycerol was associated with all pollutants and linked to myocardial infarction and stroke. Mediation analysis showed NOx’s effect on MACE was mediated by choline.

**Conclusions:**

Air pollution links to metabolic changes that contribute to cardiovascular disease progression and MACE.

Coronary artery disease (CAD) is affecting around 20.5 million adult Americans, resulting in substantial health care costs.^1^ As the leading cause of death in the United States, CAD is responsible for around 610,000 deaths each year, roughly 1 in 4 deaths.^2^ Air pollution has been recognized as a significant environmental risk factor for cardiovascular diseases, including CAD.^3^^,^^4^ Specifically, exposures to fine particulate matter (particles ≤2.5 μm in diameter) (PM_2.5_), nitrogen oxide (NOx), and carbon monoxide (CO) are well-established risk factors for CAD,^5^, ^6^, ^7^, ^8^, ^9^ possibly due to inflammation, clot formation, apoptosis, stress response, and fatty acid metabolism,^10^, ^11^, ^12^, ^13^ all of which contribute to atherosclerosis and CAD progression.^14^, ^15^, ^16^, ^17^, ^18^ However, the precise molecular mechanisms underlying these processes, and the granular role of specific air pollutants in cardiovascular risk, remain unclear. This uncertainty is largely due to the diverse and heterogeneous nature of air pollution mixtures, with varying effects on cardiovascular disease risk that complicate efforts to fully understand its impact.^19^^,^^20^

Metabolomics offers a powerful approach for studying the complete set of metabolites in biological systems, providing valuable insights into the biochemical processes and pathways influenced by environmental factors such as air pollution.^21^, ^22^, ^23^ It was shown that air pollution exposure alters levels of metabolites linked to atherosclerosis,^24^, ^25^, ^26^ contributing to the development of atherosclerotic plaques, thus exacerbating cardiovascular risk. However, the specific mediating mechanisms by which these metabolites contribute to the effects of air pollution on CAD prognosis are not yet fully understood. There is a critical need for studies that integrate high-resolution metabolomics with spatiotemporally resolved exposure data to better understand how perturbations in the air pollution–related molecular signatures and pathways contribute to CAD progression and adverse cardiovascular events. Addressing this knowledge gap could reveal novel biomarkers and potential therapeutic targets, ultimately improving CAD prognosis and public health interventions related to air quality.

## Materials and methods

### Study population

The study was conducted using data from the Emory Cardiovascular Biobank (EmCAB), which is an ongoing prospective study established in 2003, with 8,278 participants enrolled as of November 2024. The study design and cohort profile have been described previously.^27^ Briefly, patients aged 18 years and older undergoing cardiac catheterization at 3 Emory health care locations in Atlanta were invited to participate. Patients with congenital heart disease, severe valvular heart disease, severe anemia, recent blood transfusion, myocarditis, active inflammatory disease, or cancer were excluded. Follow-up assessments were performed for adverse outcomes through phone interviews, medical record reviews, state records, and the Social Security Death Index.^27^ To determine the cause of death, we used medical records and communicated with the participant’s family. The primary adverse outcomes analyzed were major adverse cardiovascular events (MACE) and all-cause death. MACE was a composite outcome of incident cardiovascular death, incident congestive heart failure, incident myocardial infarction (MI), and incident stroke. Cardiovascular death was adjudicated by cardiologists blinded to study data, defined as ischemic cardiovascular-related mortality, including fatal stroke, MI, or sudden death attributed to unknown or presumed cardiac causes in at-risk individuals. The study received approval from Emory University Institutional Review Board in Atlanta, Georgia, United States. All participants provided written informed consent at enrollment. Participants with both air pollution data and metabolomic profiling available and have no missing values for covariates adjusted were included in this study.

### Untargeted high-resolution metabolic profiling

The metabolic profiling for EmCAB has been described previously.^28^ Plasma samples for a subset of the EmCAB cohort including 833 participants went through untargeted high-resolution metabolomic profiling using liquid-chromatography coupled with high-resolution mass spectrometry. Under established protocol,^28^, ^29^, ^30^, ^31^, ^32^, ^33^, ^34^, ^35^ samples were analyzed using Thermo LTQ Velos Orbitrap high-resolution mass spectrometer (Thermo Fisher Scientific and C18 column chromatography (Higgins Analytical Inc., Targa, 2.1 × 10 cm) in positive ionization mode over 10 minutes, resulting mass to charge ratio values ranging 85 to 2000. Samples were analyzed in triplicates, only keeping metabolic features with Pearson correlation coefficient >0.7 across the triplicates to ensure high quality of data. The software apLCMS and xMSanalyzer^36^ were used for metabolic feature extraction.^37^ Batch effect was corrected using ComBat and surrogate variable analysis.^38^^,^^39^ Initial annotation of the metabolomics features was performed using xMSannotator,^40^ which matches with the Human Metabolome Database.^41^ Annotations with a confidence score of 3 indicate a high level of confidence, whereas a score of 2 indicates a medium level of confidence. Only annotations with a medium confidence level or higher were adopted. Further metabolite identification and confirmation of level 1 confidence^42^ was performed based on an in-house library^29^ of metabolites confirmed by comparing tandem mass spectrometry fragments with authentic standards and online spectral databases. Selected metabolic features were matched with this in-house library, allowing an mass to charge ratio difference of 10 ppm.

### Measurement of air pollutants

We estimated individual ambient exposures to PM_2.5_, NOx, and CO within 1 year before the biomonitoring. Specifically, the residential addresses were geocoded using ArcMAP and matched to a spatially ultrafine 250 m grid, census block group, and zip code tabulation area boundaries. Residential exposure to ambient air pollution (including CO, NOx, and PM_2.5_) for the study participants was then assessed at their residential address using well-established air quality models.^43^^,^^44^ This modeling procedure involved 2 major steps. First, Research LINE-source dispersion model for near-surface releases was used to estimate annual-averaged traffic-related air pollution with a high spatial resolution (250 × 250 m^2^). Separately, fusion modeling was used to integrate air pollution data from ground monitor with simulations from the Community Multiscale Air Quality model, which is a publicly available chemical transport model that simulates hourly air pollution concentrations with a 12 km spatial resolution.^45^ Finally, the spatial contrast indicated by Research LINE was ingested into the bias-corrected Community Multiscale Air Quality simulations. The final fused air quality model estimated daily ambient air pollution covering metropolitan Atlanta from 2005 to 2018 with a spatial resolution of approximately 250 × 250 m^2^.

### Statistical analysis

Patient characteristics were summarized as mean (SD) for continuous variables and frequency (percentage) for categorical variables. The individual ambient exposures to PM_2.5_, NOx, and CO were mean summarized within 1 year before enrollment and scaled by IQR.

The intensities of the metabolic features were median summarized based on values of the nonzero readings across triplicates. Features were excluded for downstream analysis if there were more than 20% zero readings across all participants. Statistical intensities were log 2 (intensity+1) transformed, mean centered and scaled by SD.

For the metabolome-wide association study (MWAS), linear regression models were employed to examine the associations between air pollutants as independent variables and metabolic features as dependent variables, adjusting for age, body mass index, sex (male vs female), race (Black vs other), smoking (ever smoking vs never smoking), and education (college and above vs high school and below). The Benjamini-Hochberg false discovery rate (FDR)^46^ method was used to correct for multiple testing, and numbers of associations with *P* value <0.05, FDR-corrected q value <0.2, and <0.05 were reported.

The associations between air pollutants and MACE, and its individual components including incident cardiovascular death, incident congestive heart failure, incident MI, and incident stroke were examined using Fine and Gray competing risk models^47^ while treating all-cause death as competing events. Cox proportional hazards model was used for all-cause death. For all Cox proportional hazards models, we evaluated the proportional hazards assumption using scaled Schoenfeld residuals and corresponding global tests and by visually inspecting plots of Schoenfeld residuals against time. These diagnostics did not indicate meaningful violations of the proportional hazards assumption. Same covariates in the MWAS of air pollutants were adjusted and additionally included hypertension, diabetes, history of MI, and estimated glomerular filtration rate calculated based on the Chronic Kidney Disease Epidemiology Collaboration (CKD-EPI) 2021 equation.^48^ Then, another MWAS was conducted for the association between metabolic features and the outcomes using similar approaches. The results for the confirmed metabolites associated with the air pollutants at FDR-corrected q value <0.2 were reported.

The metabolic features associated with air pollutants and adverse outcomes at FDR-adjusted q value <0.2 in the MWAS results were chosen as target features that entered pathway analysis in Mummichog (version 2.4.4).^49^ All metabolic features entered MWAS were used as the reference pool. A *P* value <0.05 for the pathway enrichment was considered statistically significant.

We additionally explored the mediating role of pollution-related metabolites in the link between air pollution exposure and adverse outcomes using the software High-Dimensional Mediation Analysis,^50^^,^^51^ which estimates both the indirect effects of the air pollution–related metabolites as mediators in a hierarchical manner, and provides a nuanced and structured way to investigate mediation effects. This approach is an extension of traditional mediation analysis and can handle multiple mediators to allow their independent contributions to explaining the outcome.^50^^,^^51^

Analyses were performed using R (version 4.4.0, R Foundation for Statistical Computing).

## Results

A total of 244 participants with air pollution data, metabolomics data, and nonmissing covariates were included in this study. The diagram of sample size is illustrated in Supplemental Figure 1. Over a median follow-up time of 9.8 (IQR: 4.6-12.2) years, a total of 97 (39.8%, 97/244) incident MACE events occurred, including 52 cardiovascular deaths, 42 congestive heart failure events, 38 MI events, and 7 stroke events. A total of 105 (43%, 105/244) participants died (Table 1). Baseline demographic and clinical characteristics were generally similar between participants included in the analytic subcohort and those with metabolomics data who were not included, with no meaningful differences observed (Supplemental Table 1). The median 1-year exposure to PM_2.5_, NOx, and CO in this cohort were 14.45 (IQR: 13.86-15.35) µg/m^3^, 50.94 (IQR: 30.79-66.38) ppb, and 910.73 (IQR: 711.50-1,063.54) ppb, respectively (Supplemental Table 2).

**Table 1:** Characteristics of the Study Participants in the Emory Cardiovascular Biobank (N = 244)

Age, y	65.33 (11.50)
Male (%)	152 (62.3)
Black (%)	60 (24.6)
BMI, kg/m^2^	29.34 (6.67)
Ever smoking (%)	182 (74.6)
eGFR, mL/min/1.73 m^2^	70.45 (24.04)
College or higher education (%)	142 (58.2)
Hypertension (%)	171 (70.1)
Diabetes mellitus (%)	90 (36.9)
MI history (%)	84 (34.4)
Cardiovascular death (%)	52 (21.3)
Incident congestive heart failure (%)	42 (17.2)
Incident MI (%)	38 (15.6)
Incident stroke (%)	7 (2.9)
Incident MACE (%)	97 (39.8)
All-cause death (%)	105 (43.0)

Values are mean (SD) for continuous variables and frequency (percentage) for categorical variables.

BMI = body mass index; eGFR = estimated glomerular filtration rate; MI = myocardial infarction; MACE = major adverse cardiovascular events.

The total effect of air pollution on outcomes is shown in Table 2. Per IQR higher PM_2.5_ was not statistically significantly associated with MACE (HR: 1.05; 95% CI: 0.86-1.30; *P* = 0.62) or all-cause death (HR: 1.10; 95% CI: 0.86-1.40; *P* = 0.44), although the point estimates were directionally consistent with an increased risk. Per IQR higher in CO was statistically associated with a 24% higher risk in MACE (subdistribution HR [sHR]: 1.24; 95% CI: 1.03, 1.49). Both NOx and CO demonstrated significant associations with cardiovascular mortality and congestive heart failure (Table 2).

**Table 2:** Associations Between Air Pollutants and Outcomes

Air Pollutant (per IQR Higher)	Cardiovascular Death		Incident Congestive Heart Failure		Incident MI		Incident Stroke		Incident MACE		All-Cause Death	
	sHR (95% CI)	*P* Value	sHR (95% CI)	*P* Value	sHR (95% CI)	*P* Value	sHR (95% CI)	*P* Value	sHR (95% CI)	*P* Value	HR (95% CI)	*P* Value
PM_2.5_	1.27 (0.96-1.68)	0.094	1.05 (0.71-1.55)	0.80	0.84 (0.60-1.18)	0.32	0.43 (0.13-1.48)	0.18	1.05 (0.86-1.30)	0.62	1.10 (0.86-1.40)	0.44
NOx	1.56 (1.24-1.97)	**0.00018**	1.42 (1.04-1.95)	**0.029**	0.97 (0.70-1.34)	0.84	1.00 (0.50-2.00)	0.99	1.19 (0.99-1.44)	0.068	1.14 (0.93-1.41)	0.20
CO	1.36 (1.13-1.64)	**0.0014**	1.38 (1.08-1.77)	**0.011**	1.07 (0.73-1.58)	0.73	1.31 (0.77-2.24)	0.32	1.24 (1.03-1.49)	**0.02**	1.14 (0.97-1.34)	0.10

CO = carbon monoxide; NOx = nitrogen oxides; PM_2.5_ = fine particulate matter (particles ≤2.5 μm in diameter); sHR = subdistribution HR; other abbreviation as in Table 1.

Models adjusted for age, sex, race, body mass index, smoking, education, hypertension, diabetes, history of MI, estimated glomerular filtration rate. **Bold** indicates statistically significant *P* value < 0.05.

**Figure 1:**
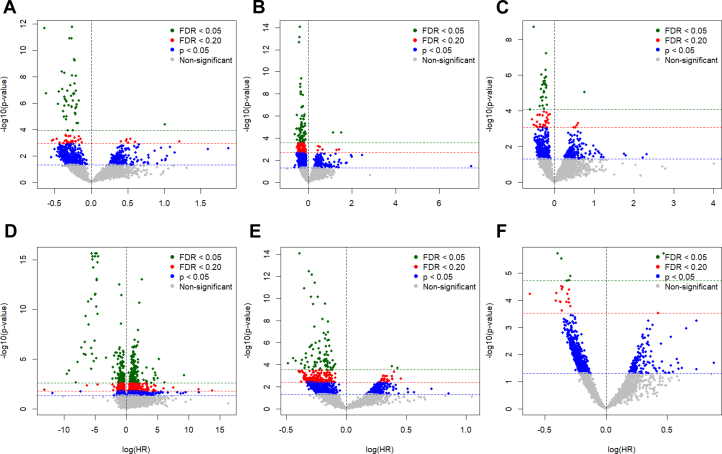
**Metabolome-Wide Association Study of the Association Between Metabolites and Outcomes** (A) cardiovascular death, (B) incident congestive heart failure, (C) incident MI, (D) incident stroke, (E) incident MACE, and (F) all-cause death. FDR = false discovery rate.

**Figure 2:**
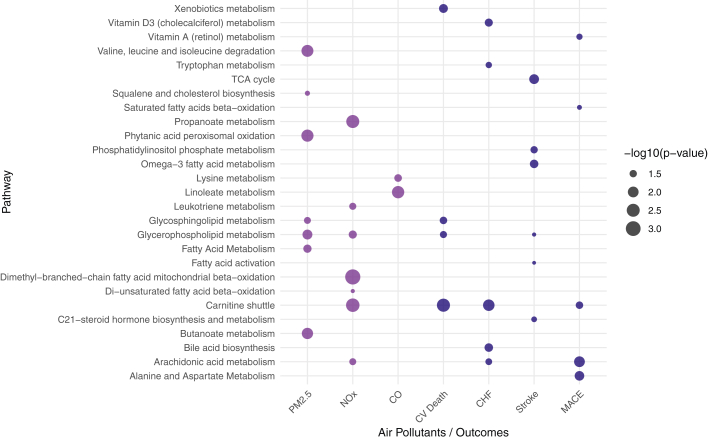
**Pathway Enrichment of Air Pollutants And Outcomes Metabolome-Wide Association Study** Metabolic features with FDR-adjusted q value <0.2 were analyzed. A *P* value <0.05 for the pathway enrichment was considered statistically significant. PM_2.5_ = fine particulate matter (particles ≤2.5 μm in diameter); NOx = nitrogen oxides; CO = carbon monoxide; CHF = congestive heart failure; MACE = major adverse cardiovascular events; CV = cardiovascular; TCA = tricarboxylic acid.

At an FDR threshold of q < 0.2, a total of 773 metabolic features were associated with PM_2.5_, 4,098 with NOx, and 533 with CO. For cardiovascular death, 82 features were linked, whereas 146 features were associated with incident congestive heart failure, 59 with incident MI, and 1,168 with incident stroke. A total of 271 features were associated with incident composite MACE, and 22 features were linked to all-cause death (Figure 1, Supplemental Table 3, Supplemental Figure 2). Statistically significant metabolic features revealed perturbations in 7, 7, and 2 enriched pathways in association with PM_2.5_, NOx, and CO, respectively (Figure 2). Glycerophospholipid metabolism was associated with both PM_2.5_ and NOx, and it was further linked to incident cardiovascular death and stroke.

The carnitine shuttle pathway was connected to metabolic features related to NOx and was enriched for features associated with incident cardiovascular death, congestive heart failure, and MACE. In addition, the arachidonic acid metabolism pathway was enriched for metabolic features linked to NOx and was associated with incident congestive heart failure and MACE (Figure 2).

Among the metabolic features associated with PM_2.5_, NOx, and CO, a total of 3, 17, and 1 metabolites had confirmed annotations based on level 1 confidence (Table 3). We identified that 1-oleoyl-rac-glycerol was positively associated with all 3 air pollutants. It was also associated with incident MI and incident stroke. Higher levels of NOx were associated with lower levels of gluconic acid (beta −0.19; 95% CI: −0.35 to −0.03), which was associated with a lower risk of MACE (sHR: 0.83; 95% CI: 0.76-0.91), cardiovascular death, congestive heart failure, and MI. Higher levels of NOx were linked to higher levels of retinoate (beta 0.18; 95% CI: 0.02-0.34), which was associated with increased risks of MACE (sHR: 1.36; 95% CI: 1.12, 1.65), cardiovascular death, and congestive heart failure. NOx was associated with higher levels of bis(2-ethylhexyl) phthalate (beta 0.18; 95% CI: 0.03-0.34), which showed associations with increased risks of MACE (sHR: 1.31; 95% CI: 1.06-1.63) and all-cause death (HR: 1.28; 95% CI: 1.05-1.57).

**Table 3:** Confirmed Metabolites Associated With Air Pollutants (FDR<0.2) and Their Associations With Outcomes

m/z	Retention Time (s)	Name	Association With Air Pollutant^a^			Association With Cardiovascular Death^b^		Association With Incident Congestive Heart Failure^b^		Association With Incident MI^b^		Association With Incident Stroke^b^		Association With Incident MACE^b^		Association With All-Cause Death^b^	
			Beta (95% CI)	*P* Value	FDR	sHR (95% CI)	*P* Value	sHR (95% CI)	*P* Value	sHR (95% CI)	*P* Value	sHR (95% CI)	*P* Value	sHR (95% CI)	*P* Value	HR (95% CI)	*P* Value
Metabolic features associated with PM_2.5_																	
118.0649	65.5	Indole	0.24 (0.1-0.37)	0.0007	0.087	1.11 (0.78-1.58)	0.56	1.02 (0.74-1.4)	0.9	1.67 (0.83-3.38)	0.15	10.39 (0.07-1,625.27)	0.36	1.23 (0.88-1.73)	0.22	1.06 (0.87-1.31)	0.55
166.0858	40.4	L-phenylalanine	0.19 (0.05-0.33)	0.0066	0.17	1.09 (0.73-1.63)	0.67	2.65 (0.83-8.45)	0.099	0.83 (0.65-1.07)	0.15	1.43 (0.51-4.03)	0.50	0.98 (0.8-1.2)	0.85	1.05 (0.83-1.33)	0.68
357.2991	539.9	1-oleoyl-rac-glycerol	0.2 (0.06-0.33)	0.0053	0.15	1.2 (0.9-1.6)	0.21	1.24 (0.92-1.66)	0.16	**1.37 (1.03-1.82)**	**0.032**	1.05 (0.48-2.31)	0.89	1.18 (0.97-1.42)	0.095	1.07 (0.88-1.29)	0.52
Metabolic features associated with NOx																	
102.0545	425.5	1-aminocyclopropane-1-carboxylate	−0.21 (−0.36 to −0.05)	0.0094	0.072	**0.73 (0.55-0.97)**	**0.029**	0.87 (0.63-1.2)	0.40	1.04 (0.72-1.51)	0.84	**0.45 (0.31-0.66)**	**0.00004**	0.85 (0.68-1.06)	0.14	0.91 (0.75-1.1)	0.31
104.1068	430.4	Choline	−0.22 (−0.37 to −0.07)	0.0054	0.055	0.81 (0.62-1.05)	0.12	**0.68 (0.5-0.93)**	**0.016**	1.06 (0.74-1.51)	0.76	0.92 (0.62-1.36)	0.66	0.81 (0.65-1.01)	0.06	0.89 (0.74-1.07)	0.21
120.0655	40.5	L-threonine; homoserine; l-allothreonine; l-threonine; homoserine; l-allothreonine	−0.16 (−0.32 to −0.01)	0.043	0.17	0.83 (0.62-1.1)	0.19	0.84 (0.63-1.13)	0.25	**0.83 (0.69-1)**	**0.048**	1.51 (0.3-7.56)	0.61	0.92 (0.77-1.1)	0.34	0.91 (0.65-1.26)	0.57
142.097	23.8	L-histidinol	−0.17 (−0.33 to −0.02)	0.031	0.13	1.02 (0.77-1.36)	0.87	0.94 (0.73-1.22)	0.65	1.16 (0.82-1.65)	0.4	1.65 (0.43-6.36)	0.47	1.01 (0.82-1.24)	0.94	0.91 (0.76-1.11)	0.36
161.1069	422.8	Tryptamine	0.2 (0.04-0.36)	0.013	0.085	1.11 (0.87-1.42)	0.4	**0.83 (0.72-0.95)**	**0.0065**	1.2 (0.79-1.84)	0.39	0.86 (0.62-1.2)	0.37	0.93 (0.82-1.06)	0.27	0.95 (0.81-1.13)	0.58
189.1597	36.1	Nepsilon, nepsilon,nepsilon-trimethyllysine	0.15 (0-0.31)	0.057	0.19	1.33 (0.94-1.9)	0.11	0.82 (0.62-1.08)	0.16	1.05 (0.71-1.57)	0.8	**0.46 (0.27-0.77)**	**0.0032**	1.03 (0.82-1.29)	0.81	**1.32 (1.05-1.66)**	**0.016**
195.0873	58.9	Methyl beta-d-galactoside; caffeine	−0.2 (−0.36 to −0.05)	0.011	0.078	1.22 (0.67-2.25)	0.51	0.84 (0.63-1.14)	0.27	1 (0.74-1.37)	0.98	0.94 (0.66-1.32)	0.70	0.9 (0.72-1.11)	0.32	1.02 (0.81-1.28)	0.88
241.0309	42	Gluconic acid	−0.19 (−0.35 to −0.03)	0.019	0.10	**0.75 (0.64-0.88)**	**0.0003**	**0.8 (0.66-0.97)**	**0.024**	**0.77 (0.67-0.88)**	**0.0001**	1.13 (0.58-2.21)	0.72	**0.83 (0.76-0.91)**	**0.00003**	0.92 (0.71-1.19)	0.52
301.2162	406.6	Retinoate	0.18 (0.02-0.34)	0.026	0.12	**1.36 (1.04-1.77)**	**0.026**	**1.38 (1.02-1.87)**	**0.038**	1.2 (0.89-1.63)	0.24	0.87 (0.3-2.53)	0.80	**1.36 (1.12-1.65)**	**0.0021**	1.19 (0.98-1.44)	0.07
344.2791	329.3	Lauroylcarnitine	−0.2 (−0.36 to −0.05)	0.012	0.079	0.9 (0.68-1.19)	0.45	0.96 (0.73-1.25)	0.76	**0.77 (0.61-0.98)**	**0.035**	1.73 (0.9-3.32)	0.099	0.89 (0.75-1.06)	0.21	1.14 (0.88-1.46)	0.32
357.2997	23.1	1-oleoyl-rac-glycerol	0.17 (0.02-0.33)	0.033	0.14	1.19 (0.86-1.64)	0.3	1.27 (0.89-1.79)	0.18	**0.68 (0.52-0.89)**	**0.0051**	**0.39 (0.23-0.64)**	**0.0002**	1.01 (0.83-1.24)	0.90	1.13 (0.92-1.39)	0.23
391.2847	530.2	Bis(2-ethylhexyl)phthalate	0.18 (0.03-0.34)	0.021	0.11	1.23 (0.93-1.64)	0.15	1.35 (0.96-1.89)	0.081	1.23 (0.87-1.72)	0.24	1.02 (0.41-2.52)	0.97	**1.31 (1.06-1.63)**	**0.014**	**1.28 (1.05-1.57)**	**0.014**
445.3116	467.6	Menaquinone	−0.26 (−0.42 to −0.11)	0.0012	0.031	0.86 (0.7-1.05)	0.13	1.06 (0.77-1.46)	0.72	0.82 (0.63-1.06)	0.13	0.67 (0.38-1.2)	0.18	0.92 (0.78-1.09)	0.33	0.9 (0.76-1.06)	0.21
563.2664	454	Protoporphyrin	−0.24 (−0.39 to −0.08)	0.0033	0.045	**0.74 (0.58-0.95)**	**0.016**	0.82 (0.63-1.08)	0.16	0.83 (0.62-1.11)	0.2	**3.8 (1.08-13.39)**	**0.037**	0.87 (0.72-1.05)	0.14	0.85 (0.7-1.03)	0.089
583.255	478.2	Biliverdin	−0.17 (−0.33 to −0.02)	0.031	0.14	0.84 (0.62-1.12)	0.23	0.85 (0.63-1.16)	0.31	0.79 (0.57-1.09)	0.15	1.61 (0.6-4.35)	0.35	0.87 (0.71-1.06)	0.16	0.97 (0.79-1.17)	0.73
777.6948	47.2	Thyroxine	−0.23 (−0.38 to −0.08)	0.0038	0.047	0.84 (0.65-1.1)	0.21	0.84 (0.61-1.15)	0.27	0.96 (0.69-1.32)	0.79	0.91 (0.38-2.21)	0.84	0.85 (0.69-1.03)	0.095	0.92 (0.76-1.12)	0.40
777.6954	227.8	Thyroxine	0.16 (0-0.31)	0.045	0.17	1.11 (0.83-1.49)	0.48	0.9 (0.64-1.26)	0.53	1.11 (0.77-1.6)	0.58	1.03 (0.48-2.24)	0.93	0.98 (0.8-1.2)	0.82	1.09 (0.89-1.33)	0.42
Metabolic features associated with CO																	
357.2997	23.1	1-oleoyl-rac-glycerol	0.17 (0.05-0.28)	0.0051	0.18	1.19 (0.86-1.64)	0.3	1.27 (0.89-1.79)	0.18	**0.68 (0.52-0.89)**	**0.0051**	**0.39 (0.23-0.64)**	**0.0002**	1.01 (0.83-1.24)	0.9	1.13 (0.92-1.39)	0.23

**Bold** indicates statistically significant *P* value < 0.05 for the association between metabolite and outcome.

FDR = false discover rate correction using the Benjamini-Hochberg procedure; m/z = mass to charge ratio; other abbreviations as in Tables 1 and 2.

^a^ Models adjusted for age, sex, race, body mass index, smoking, and education.

^b^ Models adjusted for age, sex, race, body mass index, smoking, education, hypertension, diabetes, history of MI, and estimated glomerular filtration rate.

**Figure 3:**
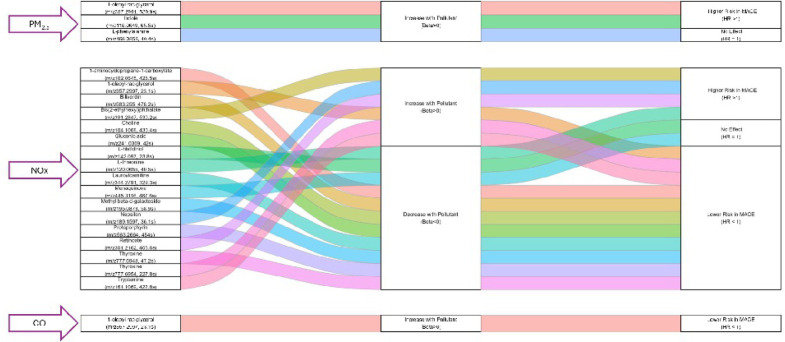
**Alluvial Plot of Mediation Between Pollutant Exposure, Metabolites, and Major Adverse Cardiovascular Events** Abbreviations as in Figure 2.

Mediation analysis revealed that higher levels of NOx are associated with lower levels of choline (beta −0.22; 95% CI: −0.38 to −0.06), which is linked with a lower risk of MACE (HR: 0.80; 95% CI: 0.65-0.98; indirect effect HR: 1.05) and congestive heart failure (HR: 0.73; 95% CI: 0.56-0.95; indirect effect HR: 1.07). Higher levels of NOx were also associated with higher levels of 1-oleoyl-rac-glycerol (beta 0.18; 95% CI: 0.02-0.34), which linked with a lower risk of MI (HR: 0.65; 95% CI: 0.47-0.90; indirect effect HR: 0.93) and stroke (HR: 0.41; 95% CI: 0.21-0.81; indirect effect HR: 0.85). In addition, higher levels of CO were associated with higher levels of 1-oleoyl-rac-glycerol (beta 0.17; 95% CI: 0.05-0.28), which linked with a lower risk of MI (HR: 0.66; 95% CI: 0.50-0.89; indirect effect HR: 0.93) and stroke (HR: 0.33; 95% CI: 0.15-0.71; indirect effect HR: 0.83) (Table 4, Figure 3).

**Table 4:** Mediation Analysis for the Confirmed Metabolites Associated With Air Pollutants (FDR<0.2) and Outcomes

m/z	Retention Time (s)	Name	Effect of Air Pollutant on Metabolite	Effect of Metabolite on Cardiovascular Death				Effect of Metabolite on Incident Congestive Heart Failure				Effect of Metabolite on Incident MI				Effect of Metabolite on Incident Stroke				Effect of Metabolite on Incident MACE				Effect of Metabolite on All-Cause Death			
			Beta (95% CI)	HR (95% CI)	Indirect Effect HR^a^	Joint P^b^	Proportion Contribution	HR (95% CI)	Indirect Effect HR^a^	Joint P^b^	Proportion Contribution	HR (95% CI)	Indirect Effect HR^a^	Joint P^b^	Proportion Contribution	HR (95% CI)	Indirect Effect HR^a^	Joint P^b^	Proportion Contribution	HR (95% CI)	Indirect Effect HR^a^	Joint P^b^	Proportion Contribution	HR (95% CI)	Indirect Effect HR^a^	Joint P^b^	Proportion Contribution
Metabolic features associated with PM_2.5_																											
118.0649	65.5	Indole	0.24 (0.1-0.38)	1.09 (0.84-1.41)	1.02	0.51	29.07	1.03 (0.77-1.38)	1.01	0.85	4.08	1.33 (0.81-2.17)	1.07	0.26	44.4	1.25 (0.62-2.51)	1.06	0.53	49.55	1.2 (0.93-1.55)	1.05	0.15	67.22	1.04 (0.87-1.25)	1.01	0.64	27.07
166.0858	40.4	L-phenylalanine	0.18 (0.04-0.32)	1.08 (0.82-1.44)	1.01	0.58	19.54	1.86 (1.05-3.29)	1.12	**0.034**	67.32	0.84 (0.66-1.09)	0.97	0.19	19.28	1.17 (0.56-2.47)	1.03	0.68	25.66	1 (0.81-1.22)	1.00	0.97	0.97	1.06 (0.87-1.3)	1.01	0.54	28.11
357.2991	539.9	1-oleoyl-rac-glycerol	0.19 (0.05-0.32)	1.23 (0.93-1.61)	1.04	0.14	51.39	1.29 (0.95-1.73)	1.05	0.099	28.6	1.35 (0.99-1.85)	1.06	0.057	36.32	1.16 (0.55-2.43)	1.03	0.70	24.8	1.12 (0.92-1.36)	1.02	0.25	31.82	1.1 (0.9-1.34)	1.02	0.34	44.82
Metabolic features associated with NOx																											
102.0545	425.5	1-aminocyclopropane-1-carboxylate	−0.21 (−0.37 to −0.05)	0.72 (0.55-0.95)	1.07	**0.021**	13.18	0.92 (0.66-1.28)	1.02	0.61	4.14	0.94 (0.68-1.3)	0.99	0.70	2.71	0.53 (0.25-1.15)	1.19	0.11	13.81	0.83 (0.67-1.03)	1.04	0.084	11.97	0.9 (0.75-1.09)	1.02	0.30	7.47
104.1068	430.4	Choline	−0.22 (−0.38 to −0.06)	0.76 (0.58-1.01)	1.06	0.057	11.82	0.73 (0.56-0.95)	1.07	**0.021**	16.32	1.01 (0.72-1.43)	1.00	0.93	0.66	0.84 (0.4-1.78)	1.03	0.65	4.11	0.8 (0.65-0.98)	1.05	**0.035**	15.27	0.9 (0.74-1.08)	1.02	0.25	8.63
120.0655	40.5	L-threonine; homoserine; l-allothreonine; l-threonine; homoserine; l-allothreonine	−0.16 (−0.32 to −0.01)	1.18 (0.78-1.8)	0.97	0.43	5.48	1.02 (0.64-1.64)	1.00	0.92	0.88	0.97 (0.7-1.33)	1.00	0.83	1.16	1.53 (0.57-4.13)	0.94	0.40	7.38	1.1 (0.87-1.4)	0.98	0.43	4.92	1.03 (0.74-1.44)	1.00	0.86	1.74
142.097	23.8	L-histidinol	−0.15 (−0.31 to 0.01)	1.02 (0.8-1.31)	1.00	0.87	0.63	0.99 (0.76-1.3)	1.00	0.97	0.2	1.09 (0.81-1.47)	0.99	0.58	2.57	1.23 (0.64-2.38)	0.97	0.53	3.3	1 (0.83-1.19)	1.00	0.97	0.17	0.93 (0.77-1.11)	1.01	0.40	4.13
161.1069	422.8	Tryptamine	0.21 (0.05-0.37)	1.03 (0.73-1.47)	1.01	0.85	1.40	0.8 (0.59-1.1)	0.95	0.17	10.6	1.12 (0.8-1.58)	1.02	0.51	4.9	0.81 (0.32-2.06)	0.94	0.66	4.61	0.92 (0.73-1.15)	0.99	0.46	5.42	0.9 (0.71-1.15)	0.98	0.40	7.62
189.1597	36.1	Nepsilon-nepsilon,nepsilon-trimethyllysine	0.14 (−0.01 to 0.3)	1.25 (0.96-1.62)	1.03	0.092	6.35	0.91 (0.67-1.23)	0.98	0.53	3.24	1.12 (0.82-1.52)	1.02	0.48	3.27	0.54 (0.29-0.98)	0.91	0.070	9.41	1.04 (0.85-1.27)	1.01	0.71	1.72	1.2 (1-1.43)	1.03	0.070	9.24
195.0873	58.9	Methyl beta-d-galactoside; caffeine	−0.21 (−0.36 to −0.05)	1.09 (0.82-1.46)	0.98	0.55	3.6	0.88 (0.69-1.13)	1.04	0.31	6.07	0.92 (0.66-1.29)	1.02	0.63	3.48	1.03 (0.5-2.09)	1.01	0.94	0.55	0.88 (0.72-1.06)	1.04	0.18	8.45	1.05 (0.84-1.3)	0.99	0.68	3.35
241.0309	42	Gluconic acid	−0.18 (−0.34 to −0.03)	0.69 (0.48-1)	1.07	0.051	13.38	0.85 (0.6-1.19)	1.04	0.33	7.21	0.79 (0.59-1.07)	1.05	0.13	8.69	1.01 (0.37-2.79)	0.97	0.98	0.21	0.84 (0.67-1.06)	1.05	0.15	9.76	0.92 (0.71-1.19)	1.02	0.51	5.77
301.2162	406.6	Retinoate	0.15 (−0.01 to 0.31)	1.25 (0.95-1.64)	1.05	0.12	6.51	1.33 (1-1.77)	1.04	0.062	10.01	1.32 (0.95-1.82)	1.01	0.093	8.51	0.9 (0.43-1.9)	0.98	0.79	1.61	1.37 (1.08-1.74)	1.05	0.062	14.66	1.13 (0.91-1.41)	1.02	0.27	6.64
344.2791	329.3	Lauroylcarnitine	−0.21 (−0.36 to −0.05)	0.89 (0.65-1.23)	1.02	0.49	4.63	1.05 (0.78-1.42)	0.99	0.75	2.38	0.76 (0.6-0.96)	1.06	**0.021**	11.63	1.29 (0.56-2.99)	0.95	0.55	5.47	0.95 (0.78-1.16)	1.03	0.63	3.16	1.09 (0.89-1.33)	0.98	0.43	6.01
357.2997	23.1	1-oleoyl-rac-glycerol	0.18 (0.02-0.34)	1.19 (0.89-1.58)	1.03	0.23	6.00	1.21 (0.88-1.66)	1.03	0.25	7.78	0.65 (0.47-0.9)	0.93	**0.029**	15.36	0.41 (0.21-0.81)	0.85	**0.029**	16.52	0.93 (0.76-1.15)	0.99	0.51	3.78	1.13 (0.93-1.38)	1.02	0.23	7.68
391.2847	530.2	Bis(2-ethylhexyl)phthalate	0.16 (0.01-0.32)	1.06 (0.8-1.4)	1.02	0.70	1.78	1.19 (0.88-1.6)	1.03	0.25	6.71	1.35 (0.98-1.86)	1.02	0.066	10.1	1.02 (0.48-2.16)	1.00	0.95	0.39	1.23 (0.98-1.54)	1.04	0.076	10.35	1.16 (0.94-1.42)	1.02	0.18	8.46
445.3116	467.6	Menaquinone	−0.27 (−0.43 to −0.11)	0.96 (0.76-1.22)	1.03	0.76	2.00	1.13 (0.86-1.48)	0.97	0.38	7.64	0.89 (0.69-1.15)	1.05	0.37	6.47	0.63 (0.31-1.27)	1.11	0.20	13.03	1.0 (0.83-1.2)	1.01	0.97	0.32	0.92 (0.76-1.1)	1.02	0.35	8.53
563.2664	454	Protoporphyrin	−0.22 (−0.38 to −0.06)	0.75 (0.55-1.02)	1.07	0.071	12.45	0.92 (0.69-1.21)	1.02	0.54	4.49	0.88 (0.65-1.18)	1.03	0.40	5.75	1.57 (0.77-3.19)	0.86	0.22	10.39	0.97 (0.78-1.22)	1.01	0.82	1.80	0.91 (0.72-1.14)	1.02	0.40	7.77
583.255	478.2	Biliverdin	−0.18 (−0.33 to −0.02)	0.84 (0.64-1.1)	1.03	0.21	5.93	0.91 (0.68-1.23)	1.02	0.55	3.72	0.8 (0.58-1.11)	1.04	0.18	7.86	1.33 (0.64-2.77)	0.95	0.45	5.27	0.96 (0.78-1.17)	1.02	0.68	2.29	1 (0.83-1.22)	1.00	0.97	0.21
777.6948	47.2	Thyroxine	−0.22 (−0.37 to −0.06)	1.06 (0.8-1.39)	1.00	0.69	2.40	1.00 (0.75-1.34)	1.00	0.99	0.13	1.09 (0.81-1.46)	0.98	0.57	3.83	0.87 (0.41-1.84)	1.04	0.72	3.16	0.96 (0.76-1.21)	1.01	0.73	2.71	1.04 (0.84-1.29)	0.99	0.72	3.03
777.6954	227.8	Thyroxine	0.13 (−0.02 to 0.29)	1.1 (0.83-1.45)	1.01	0.51	2.45	0.76 (0.56-1.04)	0.98	0.089	8.5	1.12 (0.81-1.54)	1.00	0.49	3.06	1.06 (0.51-2.21)	1.00	0.88	0.81	0.92 (0.75-1.15)	0.99	0.48	3.24	1.08 (0.88-1.33)	1.01	0.46	3.71
Metabolic features associated with CO																											
357.2997	23.1	1-oleoyl-rac-glycerol	0.17 (0.05-0.28)	1.13 (0.85-1.5)	1.02	0.41	-	1.14 (0.83-1.56)	1.02	0.44	-	0.66 (0.5-0.89)	0.93	**0.0056**	-	0.33 (0.15-0.71)	0.83	**0.0053**	-	0.94 (0.77-1.14)	0.99	0.51	-	1.1 (0.9-1.33)	1.02	0.36	-

**Bold** indicates statistically significant joint *P* value < 0.05.

Abbreviations as in Table 1, Table 2, Table 3.

Models adjusted for age, sex, race, body mass index, smoking, education, hypertension, diabetes, history of MI, and estimated glomerular filtration rate.

^a^ Indirect effect HR: beta for the effect of air pollutant on metabolite × beta (log HR) for the effect of metabolite on outcome. CI was not derived by HIMA.

^b^ Joint P: defined as the larger of the *P*-values for the air pollutant-metabolite and metabolite-outcome paths.

## Discussion

Leveraging the spatiotemporally resolved air pollution data and the advanced high-resolution metabolomics profiling, we aimed to elucidate the mechanisms underlying the toxicity of air pollutants on cadiovascular disease in the well-established EmCAB. To the best of our knowledge, it is the first study of its kind on the association between air pollution exposure, metabolic alterations, and cardiovascular disease risk. We also investigated the potential mediating role of specific metabolites in the relationship between air pollutants and adverse cardiovascular outcomes. Our findings suggest that exposure to air pollution, specifically PM_2.5_, NOx, and CO, is strongly associated with metabolic variations that may contribute to the progression of CAD and adverse cardiovascular events (Central Illustration).

**Central Illustration:**
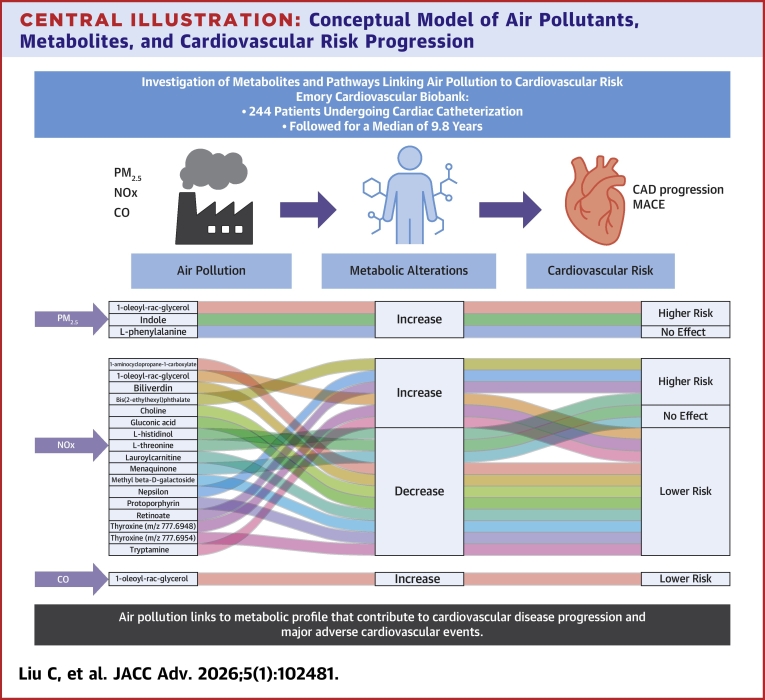
**Conceptual Model of Air Pollutants, Metabolites, and Cardiovascular Risk Progression** The top section presents a conceptual model illustrating how exposure to air pollutants (PM_2.5_, NOx, and CO) leads to metabolomic alterations, which in turn mediate the progression of coronary artery disease and major adverse cardiovascular events. The lower section provides a detailed visualization of the specific metabolites involved. It shows the associations between each pollutant and a set of metabolites and the corresponding impact on cardiovascular risk. PM_2.5_ = fine particulate matter (particles ≤2.5 μm in diameter); NOx = nitrogen oxides; CO = carbon monoxide; MACE = major adverse cardiovascular events; CAD = coronary artery disease.

Previous studies of airborne PM_2.5_ exposures have revealed its association with cardiovscular risk.^8^ A study reported that PM_2.5_ exposure affected pathways related to fatty acid metabolism,^10^ linked to the development of atherosclerosis,^16^ a key causal factor in CAD.^15^ NOx is an established risk factor in cardiovascualr diseases,^5^, ^6^, ^7^ with studies showing associations between NOx and platelet counts and plasma fibrinogen, suggesting that NOx may contribute to cardiovascular risk by promoting inflammation and clot formation, both of which play a significant role in atherosclerosis and thrombosis.^17^^,^^18^ Previous studies have shown that CO is a complex exposure with both harmful and potentially protective effects on the cardiovascular system.^9^ High levels of CO, which significantly increase carboxyhemoglobin, are known to have cardiotoxic effects.^9^ High CO exposure reduces the oxygen-carrying capacity of arterial blood, negatively impacting aerobic capacity and exercise performance.

We found that gluconic acid was negatively associated with NOx, and we observed negative associations between gluconic acid and adverse cardiovascular outcomes. Gluconic acid, a product of enzyme glucose oxidase activity, has been associated with oxidative stress and is recognized as an oxidative stress-related metabolite. A previous study found that gluconic acid was associated with hyperglycemia and cytotoxic brain injury after stroke.^52^ Further studies are needed to elucidate the biological pathways linking air pollution to gluconic acid levels, including investigations into enzymatic regulation, metabolic adaptations, and potential interactions with other oxidative stress markers. In addition, retinoate, a metabolite of retinol (vitamin A), was found to be positively associated with NOx, which in turn was linked to adverse cardiovascular outcomes. The observed positive relationship between retinoate and cardiovascular outcomes aligns well with previous findings, where excessive serum vitamin A levels were associated with an increased risk of cardiovascular mortality and mortality related to CAD in the National Health and Nutrition Examination Survey (NHANES) cohort.^53^

Notably, mediation analysis provided novel insights into the potential mechanistic pathways through which air pollution may influence cardiovascular outcomes. Specifically, we observed that elevated NOx levels were associated with lower levels of choline, which, in turn, linked with a reduced risk of MACE and congestive heart failure. Choline, an essential metabolite for cell membrane formation, normal liver and kidney functions, and neurotransmitter production,^54^ was negatively associated with NOx exposure, a finding consistent with previous studies that have reported metabolomic perturbations following PM_2.5_ and NOx exposure.^55^ Furthermore, choline was also negatively associated with the incident congestive heart failure and MACE. The study of NHANES found a significant inverse association between dietary choline intake and cardiovascular risk,^54^ further suggesting that choline may serve as a protective mediator for the cardiovascular system and may be reduced following exposure to air pollution. Taken together, these findings suggest that the disruption of choline metabolism by air pollution could play an important role in exacerbating cardiovascular risk, potentially offering a target for future therapeutic strategies.

Our study has several strengths. First, we leveraged the well-established EmCAB cohort, which provides comprehensive clinical data and reliable assessments of cardiovascular disease outcomes. Second, the use of advanced high-resolution metabolomics allows for a comprehensive analysis of metabolic alterations associated with air pollution exposure. In addition, our application of spatiotemporally resolved air pollution models ensures precise and individualized exposure assessments, enhancing the validity of our findings. Finally, the innovative use of high-dimensional mediation analysis enabled us to uncover complex mediation pathways, providing novel insights into the molecular mechanisms linking air pollution to cardiovascular disease risk. These strengths collectively contribute to the robustness and significance of our study.

### Study Limitations

Despite these promising findings, our study has limitations. First, although we adjusted for key demographic and clinical covariates, factors such as medication, diet, physical activity, or unmeasured environmental exposures could influence both the metabolome and cardiovascular outcomes. Our exposure modeling incorporated traffic intensity and multiple area-level indicators (social deprivation, greenspace, and health care access), and individual-level education was included as a covariate. Further adjustment for these same factors in health outcome models would represent overadjustment. Nevertheless, residual neighborhood-level confounding cannot be fully excluded and should be considered a limitation. Second, although we had 97 MACE events, providing reasonable power for analyses of the composite outcome after adjusting for covariates, the number of events for individual outcomes such as heart failure, MI, and particularly stroke was limited. This raises the possibility of reduced statistical power and unstable effect estimates for these outcomes. Although some pollutant-MACE associations did not reach statistical significance in our cohort, mediation analysis does not require a significant total effect to provide meaningful insights. Given the well-established evidence linking air pollution to adverse cardiovascular outcomes,^56^ our mediation results are best interpreted as exploratory mechanistic insights into potential biological pathways rather than as a redemonstration of the exposure-outcome relationship. Third, only a subset of metabolites reached level 1 confirmation. Our pathway analyses leveraged Mummichog,^49^ which is specifically designed to extract pathway-level insights from untargeted metabolomics data where many features remain at putative annotation levels. Nevertheless, limited metabolite validation may restrict biological interpretability, and future studies incorporating expanded targeted validation will be important to strengthen reproducibility. Fourth, air pollution exposure was estimated based on the residential address of the participants, which did not consider the daily mobility or exposures in the indoor and other microenvironment. This approach may not accurately reflect personal exposures, as many individuals spend a substantial amount of time indoors,^57^ potentially resulting in exposure misclassification.^58^ In addition, since information on participants’ time-activity patterns and microenvironmental characteristics was not available, we were unable to incorporate indoor/outdoor (I/O) concentration ratios into the exposure assessment. Applying a uniform I/O ratio across the cohort would not meaningfully alter exposure rankings and would require strong assumptions about individual behaviors. Thus, the lack of I/O information may contribute to some exposure misclassification. Fifth, another important consideration in our study is the potential effect of nonfasting samples on the metabolome. The participants included in this study were not required to fast before sample collection, which could introduce variability due to diet and nutritional intake at the time of sample collection. Sixth, because EmCAB recruited patients referred for cardiac catheterization, our findings may not be generalizable to the broader population. This design could in principle introduce collider bias if both air pollution exposures and metabolomic signatures influence the likelihood of undergoing catheterization. However, as our analyses focus on associations within this clinical cohort rather than estimating population-level incidence, such bias is less likely to materially affect the validity of our findings. Seventh, our exposure assessment focused on 1-year average air pollution before enrollment, consistent with prior metabolomics literature indicating that most metabolomic signals reflect short-to medium-term exposures.^59^ Although longer-term or time-varying exposure windows could provide additional insights, our exposure models (2005-2018) and cohort recruitment period (2004-2011) would reduce the sample size substantially, limiting statistical power. Future work with extended exposure modeling and repeated biospecimens will be important to capture long-term dynamics. Finally, our mediation analysis should be interpreted with caution, as the modest sample size may lead to unstable indirect-effect estimates, and residual or unmeasured confounding may still be present. These findings therefore require validation in larger, independent cohorts. We were unable to perform validation in an independent data set or conduct a split-sample analysis due to the modest sample size; however, given the exploratory and hypothesis-generating nature of this study, our findings provide a foundation for future replication efforts.

## Conclusions

Our findings suggest that air pollution exposure may lead to alterations in metabolite profiles that in-turn mediate the increased risk of cardiovascular events. The identification of specific metabolites offers valuable, novel insights into the molecular mechanisms underlying the cardiovascular effects of air pollution and highlights potential biomarkers for early detection and intervention. Further research into the mechanisms identified in this study may lead to novel therapeutic targets aimed at mitigating the cardiovascular impact of air pollution, ultimately improving public health outcomes related to both air quality and cardiovascular disease.

Perspectives **COMPETENCY IN MEDICAL KNOWLEDGE:** Health care providers should integrate air pollution exposure as a significant cardiovascular risk factor in patient evaluations. This awareness can guide preventive measures, such as recommending air quality improvements and lifestyle modifications for patients in high-risk environments. Clinicians should consider how pollutants like PM_2.5_, NOx, and CO influence metabolic alterations linked to cardiovascular disease, particularly in vulnerable populations.

**TRANSLATIONAL OUTLOOK:** This study provides a foundation for using metabolic biomarkers to assess cardiovascular risk in patients exposed to air pollution. These metabolites may serve as early indicators, enabling more targeted interventions to reduce cardiovascular disease progression. Further research and clinical validation are needed to translate these findings into practical tools for risk assessment and therapeutic strategies in clinical settings.

## Funding support and author disclosures

The study was supported by the pilot award through the Emory Human Exposome Research Center (HERCULES), supported by the 10.13039/100000066National Institute of Environmental Health Sciences of the 10.13039/100000002National Institutes of Health under Award Number P30ES019776. Research reported in this publication was also supported by the 10.13039/100000002National Institutes of Health (NIH) research grant (R01ES035738). The content of this publication is solely the responsibility of the authors and does not necessarily represent the official views of the National Institutes of Health. The authors have reported that they have no relationships relevant to the contents of this paper to disclose.
