# Supplementary material for: Metabolic Signatures of Air Pollution and Major Adverse Cardiovascular Events in Patients Undergoing Cardiac Catheterization
Source: JACC Adv. 2026 Jan 28;5(1):102481. doi: 10.1016/j.jacadv.2025.102481 (PMC12869874; doi:10.1016/j.jacadv.2025.102481)

**Table S1**. Characteristics of the study participants in the Emory Cardiovascular Biobank, stratified by inclusion.

| **Characteristics** | **Included**  **(N = 244)** | **With Metabolomics Data, Not Included**  **(N = 589)** | **P** | **Bonferroni Corrected P < 0.05** |
| --- | --- | --- | --- | --- |
| **Age, years** | 65.33 (11.50) | 66.80 (11.03) | 0.085 | No |
| **Male (%)** | 152 (62.3) | 391 (66.4) | 0.24 | No |
| **Black (%)** | 60 (24.6) | 69 (11.7) | <0.001 | Yes |
| **BMI, kg/m^2^** | 29.34 (6.67) | 28.43 (4.86) | 0.031 | No |
| **Ever Smoking (%)** | 182 (74.6) | 367 (68.2) | 0.085 | No |
| **eGFR, mL/min/1.73m^2^** | 70.45 (24.04) | 67.40 (23.76) | 0.094 | No |
| **College or Higher Education (%)** | 142 (58.2) | 263 (48.5) | 0.015 | No |
| **Hypertension (%)** | 171 (70.1) | 415 (71.1) | 0.842 | No |
| **Diabetes Mellitus (%)** | 90 (36.9) | 233 (39.8) | 0.486 | No |
| **MI History (%)** | 84 (34.4) | 189 (32.3) | 0.599 | No |
| **Cardiovascular Death (%)** | 52 (21.3) | 174 (31.3) | 0.005 | No |
| **Incident Congestive Heart Failure (%)** | 42 (17.2) | 99 (17.8) | 0.023 | No |
| **Incident MI (%)** | 38 (15.6) | 76 (13.7) | 0.035 | No |
| **Incident Stroke (%)** | 7 (2.9) | 32 (5.8) | 0.006 | No |
| **Incident MACE (%)** | 97 (39.8) | 258 (46.4) | 0.128 | No |
| **All-cause Death (%)** | 105 (43.0) | 302 (54.3) | 0.004 | No |

Abbreviations: BMI, Body Mass Index; eGFR, estimated glomerular filtration rate; MI, myocardial infarction; MACE, major adverse cardiovascular events.

**Table S2.** Summary of air pollutant exposures among participants.

| **Pollutant** | **Unit** | **Minimum** | **Median (IQR)** | **Mean (SD)** | **Maximum** |
| --- | --- | --- | --- | --- | --- |
| PM_2.5_ | ug/m^3^ | 10.12 | 14.45 (13.86-15.35) | 14.44 (1.38) | 18.70 |
| NOx | ppb | 14.08 | 50.94 (30.79-66.38) | 53.34 (29.61) | 201.35 |
| CO | ppb | 367.83 | 910.73 (711.50-1063.54) | 955.05 (394.66) | 3018.42 |

Abbreviations: IQR, interquartile range; Ppb, parts per billion; SD, standard deviation; PM_2.5_, fine particulate matter; NOx, nitrogen oxides; CO, carbon monoxide.

**Table S3.** Metabolic features significantly associated with air pollutants and outcomes.

| **Statistical Significance Threshold** | **Number of Metabolic Features** | |
| --- | --- | --- |
| **Associated with Air Pollutant*** | | |
| **P < 0.05** | **PM_2.5_** | 1904 |
|  | **NOx** | 3744 |
|  | **CO** | 1936 |
| **FDR Corrected q < 0.2** | **PM_2.5_** | 773 |
|  | **NOx** | 4098 |
|  | **CO** | 533 |
| **FDR Corrected q < 0.05** | **PM_2.5_** | 17 |
|  | **NOx** | 1153 |
|  | **CO** | 15 |
| **Associated with Cardiovascular Death**** | | |
| **P < 0.05** | 1269 | |
| **FDR Corrected q < 0.2** | 82 | |
| **FDR Corrected q < 0.05** | 47 | |
| **Associated with Incident Congestive Heart Failure**** | | |
| **P < 0.05** | 1101 | |
| **FDR Corrected q < 0.2** | 146 | |
| **FDR Corrected q < 0.05** | 71 | |
| **Associated with Incident MI**** | | |
| **P < 0.05** | 662 | |
| **FDR Corrected q < 0.2** | 59 | |
| **FDR Corrected q < 0.05** | 28 | |
| **Associated with Incident Stroke**** | | |
| **P < 0.05** | 1736 | |
| **FDR Corrected q < 0.2** | 1168 | |
| **FDR Corrected q < 0.05** | 719 | |
| **Associated with Incident MACE**** | | |
| **P < 0.05** | 1560 | |
| **FDR Corrected q < 0.2** | 271 | |
| **FDR Corrected q < 0.05** | 82 | |
| **Associated with All-cause Death**** | | |
| **P < 0.05** | 1161 | |
| **FDR Corrected q < 0.2** | 22 | |
| **FDR Corrected q < 0.05** | 6 | |

Abbreviations: PM_2.5_, fine particulate matter; NOx, nitrogen oxides; CO, carbon monoxide; FDR, false discover rate correction using the Benjamini-Hochberg procedure; MI, myocardial infarction; MACE, major adverse cardiovascular events.

*Models adjusted for age, sex, race, body mass index, smoking, education.

**Models adjusted for age, sex, race, body mass index, smoking, education, hypertension, diabetes, history of MI, estimated glomerular filtration rate.

**Figure S1.** Sample size flowchart.

**
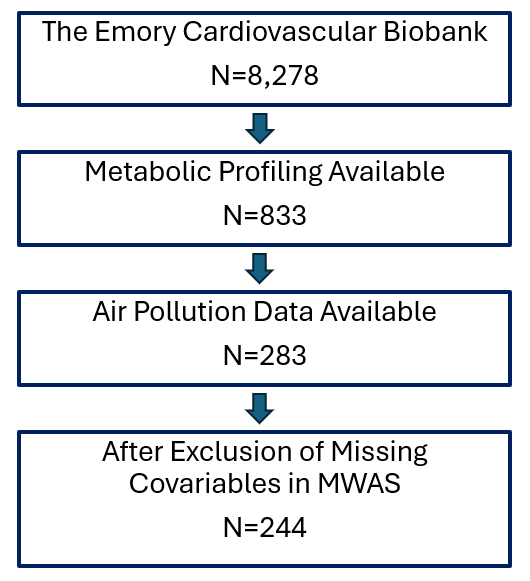
**

**Figure S2.** Metabolome-wide association study of the association between air pollution and metabolites. A: PM_2.5_; B: NOx; C: CO.


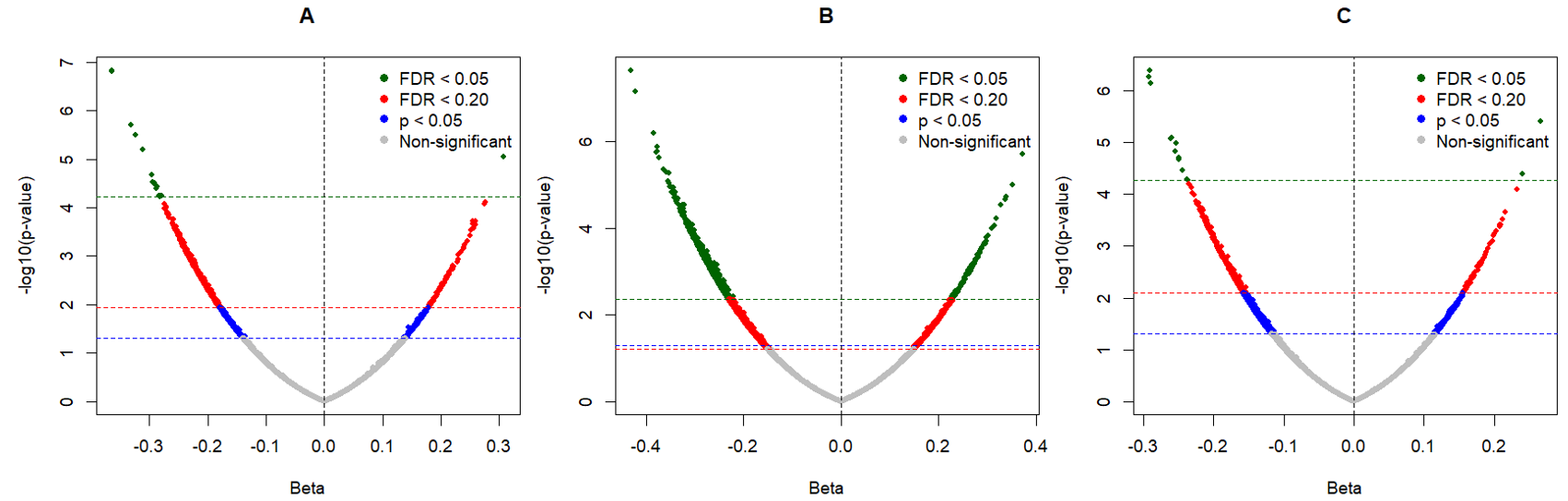

Supplement: Supplemental_Material [file mmc1.docx]
